# Supplementary material for: Comprehensive characterization of nonlinear viscoelastic properties of arterial tissues using guided-wave optical coherence elastography
Source: ArXiv. 2025 Jul 27:arXiv:2507.20107v1. Preprint. [Version 1] (PMC12310123)
Supplement: 1 [file NIHPP2507.20107v1-supplement-1.pdf]

Comprehensive characterization of nonlinear viscoelastic properties of arterial tissues using guided-wave optical coherence elastography

Yuxuan Jiang [1, †], Guo-Yang Li [1, 5, †], Ruizhi Wang [3, †], Xu Feng [1, 6, †],

Yanhang Zhang [3, 4, *], Seok-Hyun Yun [1, 2, *]

[1] Harvard Medical School and Wellman Center for Photomedicine, Massachusetts General Hospital, Boston, MA 02114, USA

[2] Harvard-MIT Health Sciences and Technology, Cambridge, MA 02139, USA

[3] Department of Mechanical Engineering, Boston University, Boston, MA 02215, USA

[4] Department of Biomedical Engineering, Boston University, Boston, MA 02215, USA

[5] Currently with the Department of Mechanics and Engineering Science, College of Engineering, Peking University, Beijing 100871, China

[6] Currently with the Department of Bioengineering, University of Texas at Dallas, TX 75080, USA

[†] Co-first authors with equal contribution.

* Corresponding authors: yanhang@bu.edu (Y.Z.), syun@hms.harvard.edu (S.H.Y.).

Supplementary Figures

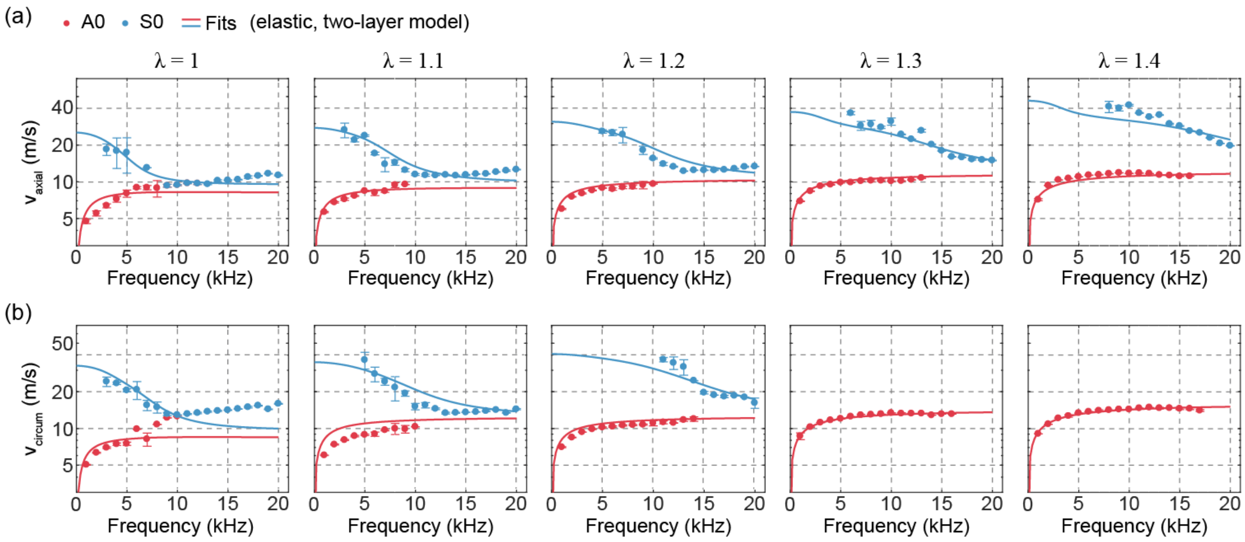

Figure S1. **Two-layer elastic model analysis of experimental data.** (a) Axial dispersion relations of A0 and S0 modes. (b) Circumferential dispersion relations of A0 and S0 modes. Markers: experiments. Lines: fitting curves using the bitwo-layer elastic model. The secular equation of the bitwo-layer elastic model is given by Eq. (S28) in Supplementary Note 4. The fitting parameters are listed in Supplementary Table S1.

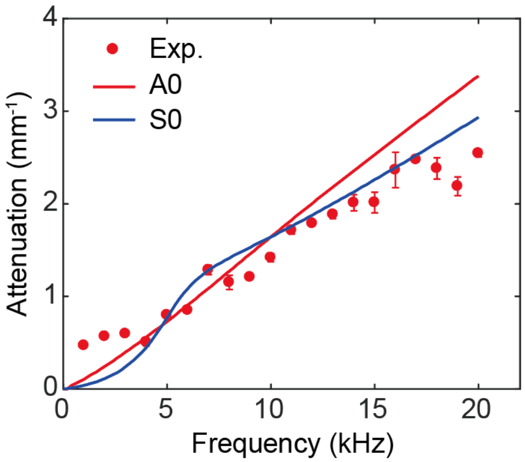

Figure S2. Wave attenuation in the axial direction at λ = 1. Markers: experiments. Lines: two-layer viscoelastic model-predicted wave attenuation for the A0 mode (red) and S0 mode (blue). Material parameters are provided in Table 3.

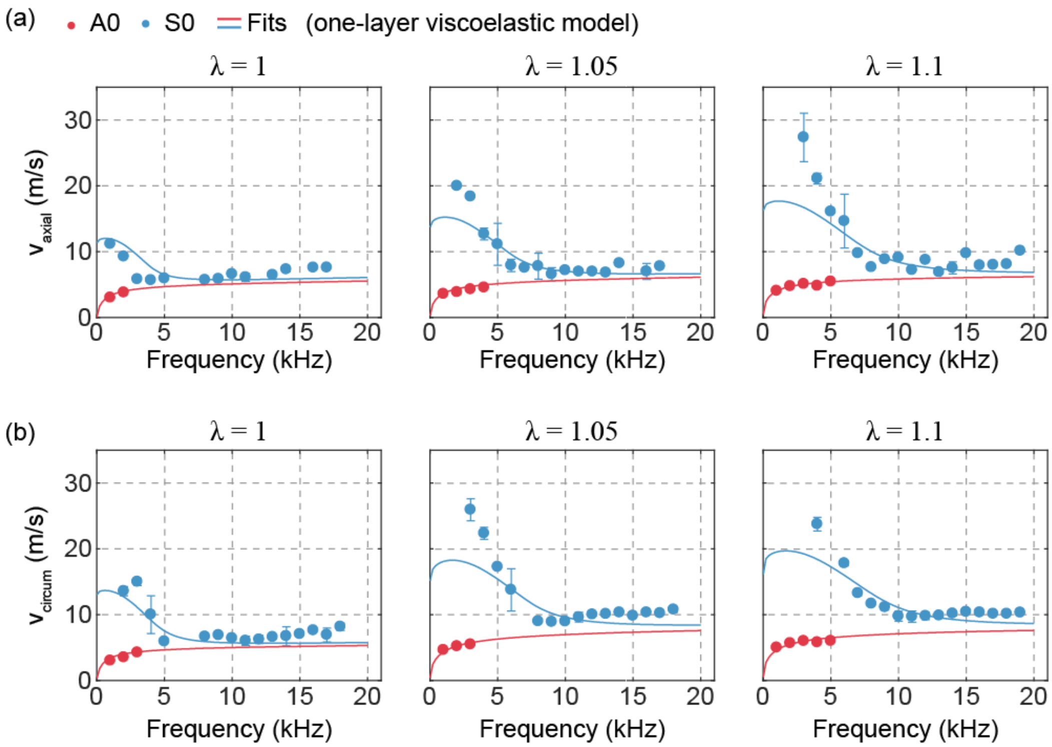

Figure S3. Experimental dispersion of the artery after CNBr treatment. (a) Axial data, (b) Circumferential data. Markers: experiments. Lines: fitting curves using the one-layer viscoelastic model.

Supplementary Note 1. Derivation of the pre-stressed elastic single-layer model

Consider an elastic material that is subjected to finite deformation, and infinitesimal elastic waves are superimposed on the static deformation. The equation of wave motion is [1]

$$\nabla \cdot \boldsymbol{\Sigma} = \rho \boldsymbol{u}_{,tt}, \tag{S1}$$

where $\boldsymbol{\Sigma}$ denotes incremental stress induced by elastic waves. $\boldsymbol{u}$ denotes the displacement of wave motion. ρ denotes the density of material. *t* denotes the time. The subscript with a comma denotes partial differentiation with respect to the corresponding variable. The harmonic elastic wave can be described as $\boldsymbol{u} = \boldsymbol{u}_0 \exp(i(\boldsymbol{k} \cdot \boldsymbol{x} - \omega t))$. where $\boldsymbol{u}_0$, $\boldsymbol{k}$ and ω denote wave amplitude, wave vector, and angular frequency, respectively. For incompressible elastic materials, the incremental stress $\boldsymbol{\Sigma}$ is related to the displacement by

$$\Sigma_{ij} = \mathcal{A}^0_{ijkl} u_{l,k} - \hat{p}\delta_{ij} + p u_{i,j}, \qquad i,j,k,l \in \{x,y,z\} \tag{S2}$$

where $\hat{p}$ denotes the increment of the Lagrange multiplier p. $\mathcal{A}^0_{ijkl}$ is the fourth-order Eulerian elasticity tensor defined as [1,2]

$$\mathcal{A}^0_{ijkl} = F_{iI} F_{kJ} \frac{\partial^2 W}{\partial F_{jI} \partial F_{lJ}}, \qquad i,j,k,l,I,J \in \{x,y,z\} \tag{S3}$$

where $\boldsymbol{F} = \mathrm{diag}(\lambda_x, \lambda_y, \lambda_z)$ is the deformation gradient tensor and W is the strain energy function of the material. Inserting Eqs. (S2) and (S3) into Eq. (S1), the wave equation is [1,2]

$$-\hat{p}_{,j} + \mathcal{A}^0_{ijkl} u_{l,ik} = \rho u_{j,tt}, \qquad i,j,k,l \in \{x,y,z\} \tag{S4}$$

Consider a flat plate with top side in contact with air and the bottom with fluid. The wall thickness of the plate is denoted as h. A Cartesian coordinate system (*x*, *y*, *z*) was established on the plate, where the *y*-axis denotes the thickness direction, and the *x*- and *z*-axes lies within the plane of the plate. The plate is subjected to in-plane biaxial stretch, with stretch ratios λ_x and λ_z. Without loss of generality, we assume that waves in the plate propagate along the *x* direction, with displacement components confined to the *x*-*y* plane (i.e., $u_z = 0$), the stream function

$\psi(x, y, t)$ can be used to replace displacements: $u_x = \psi_{,y}$ and $u_y = -\psi_{,x}$. Inserting ψ into Eq. (S4), the wave equation becomes

$$\alpha\psi_{,xxxx} + 2\beta\psi_{,xxyy} + \gamma\psi_{,yyyy} = \rho(\psi_{,xxtt} + \psi_{,yytt}), \tag{S5}$$

where α, β, and γ are acoustoelastic parameters defined by $\alpha = \mathcal{A}^0_{xyxy}$, $2\beta = \mathcal{A}^0_{xxxx} + \mathcal{A}^0_{yyyy} - 2\mathcal{A}^0_{xxyy} - 2\mathcal{A}^0_{xyyx}$ and $\gamma = \mathcal{A}^0_{yxyx}$. Explicit forms of α, β, and γ are given in Supplementary Note 5. To solve Eq. (S5), a harmonic form of the stream function is assumed: $\psi = \psi_0 \exp(sky) \exp[i(kx - \omega t)]$, where ψ_0 is an amplitude; s is a complex decay parameter; ω $(= 2\pi f)$ is the angular frequency; k is the complex wave number. Inserting the harmonic form of ψ into Eq. (S5) yields

$$\gamma s^4 - \left(2\beta - \rho\frac{\omega^2}{k^2}\right)s^2 + \alpha - \rho\frac{\omega^2}{k^2} = 0. \tag{S6}$$

Four roots, denoted as $\pm s_1$ and $\pm s_2$, can be obtained from Eq. (S6). Thus, the general solution of ψ takes the form: $\psi = \sum_{i=-2}^{2} \psi_i \, e^{sign(i) s_i k y} e^{i(kx - \omega t)}$.

The semi-infinite fluid at the bottom of the plate exhibits no rotational motion during the propagation of linear elastic waves, therefore a potential function ϕ is introduced to describe displacements of the fluid: $u_x^f = \phi_{,x}$, $u_y^f = \phi_{,y}$. The governing equation for acoustic waves in an inviscid stationary fluid is

$$\phi_{,xx} + \phi_{,yy} = \frac{1}{c_f^2}\phi_{,tt} \tag{S7}$$

where c_f $(= \sqrt{\kappa_f/\rho_f})$ is the sound speed in the fluid. κ_f denotes bulk modulus of the fluid. ρ_f denotes the fluid density. The potential function also follows a harmonic form of $\phi = \phi_0 \exp(\xi k y) \exp[i(kx - \omega t)]$. ξ can be solved by inserting ϕ into Eq. (S7), which yields

$$\xi^2 = 1 - \frac{1}{c_f^2}\frac{\omega^2}{k^2} \tag{S8}$$

The top surface of the plate exposed to air (at *y* = *h*) is stress free, and the bottom surface of

the plate in contact with fluid (at y = 0) ensures continuity of normal displacement and stress. These boundary conditions can be written as [3]:

$$u_y = u_y^f,\ \Sigma_{yx} = 0, \Sigma_{yy} = -p^f, \text{at}\ \ y = 0;$$
$$\Sigma_{yx} = 0, \Sigma_{yy} = 0,\ \text{at}\ \ y = h, \tag{S9}$$

where u_i and u_i^f denote the displacement of the plate and the fluid, respectively. Σ_{ij} denotes the incremental stress of the plate. p^f is the hydrostatic pressure of the fluid. With boundary conditions Eq. (S9), and replacing $\boldsymbol{u}$, $\boldsymbol{u}^{\mathrm{f}}$, $\boldsymbol{\Sigma}$ and p^f with ψ and φ, we obtain the secular equation of the elastic single-layer model as follows

$$\det(\mathbf{M}_{\mathbf{5}\times\mathbf{5}}^{\mathrm{e}}) = 0 \tag{S10}$$

where the components of the matrix $\mathbf{M}_{\mathbf{5}\times\mathbf{5}}^{\mathrm{e}}$ are

$M_{11} = M_{12} = M_{13} = M_{14} = 1, M_{15} = -i\xi,$

$M_{21} = 1 + s_1^2, M_{22} = 1 + s_1^2, M_{23} = 1 + s_2^2, M_{24} = 1 + s_2^2, M_{25} = 0,$

$M_{31} = \gamma s_1(1 + s_2^2), M_{32} = -\gamma s_1(1 + s_2^2), M_{33} = \gamma s_2(1 + s_1^2), M_{34} = -\gamma s_2(1 + s_1^2), M_{35} = i\rho_f\omega^2/k^2,$

$M_{41} = (1 + s_1^2)\exp(s_1kh), M_{42} = (1 + s_1^2)\exp(-s_1kh),$

$M_{43} = (1 + s_2^2)\exp(s_2kh),\ M_{44} = (1 + s_2^2)\exp(-s_2kh), M_{45} = 0,$

$M_{51} = s_1(1 + s_2^2)\exp(s_1kh), M_{52} = -s_1(1 + s_2^2)\exp(-s_1kh),$

$M_{53} = s_2(1 + s_1^2)\exp(s_2kh), M_{54} = -s_2(1 + s_1^2)\exp(-s_2kh), M_{55} = 0.$

(S11)

where i in the element M_{15} and M_{35} denotes the imaginary unit.

Supplementary Note 2. Derivation of the pre-stressed viscoelastic single-layer model

Consider a pre-stressed viscoelastic material subjected to linear elastic wave propagation; the wave equation still follows Eq. (S1). However, since the constitutive relation of the material has changed, the incremental stress for the incompressible viscoelastic material is given by [4]

$$\Sigma_{ij} = -\hat{q}\delta_{ij} + qu_{i,j} - G\hat{Q}\delta_{ij} + GQu_{i,j} + G\mathcal{A}^0_{ijkl}u_{l,k} - \Omega\sigma^e_{Dik}u_{j,k}, \quad i,j,k,l \in \{x,y,z\} \tag{S12}$$

where $\hat{q}$ denotes the increment of the Lagrange multiplier q. $\hat{Q}$ is the increment of the volumetric part of the elastic stress Q $(= \sigma^e_{ii}/3$, summation with respect to i). The elastic Cauchy stress $\boldsymbol{\sigma}^e = (\partial W/\partial \boldsymbol{F})\boldsymbol{F}^{\mathrm{T}}$. $\mathcal{A}^0_{ijkl}$ is the fourth-order Eulerian elasticity tensor defined by Eq. (S3). G and Ω in Eq. (S12) are two frequency-dependent parameters,

$$G = 1 + \eta(i\omega)^\delta,\ \ \Omega = \eta(i\omega)^\delta. \tag{S13}$$

where η (unit s^δ) and δ (0<δ<1, unit 1) are two viscoelastic parameters of the Kelvin-Voigt fractional derivative (KVFD) model [5,6]. η denotes the ratio of material viscosity to elasticity. δ is a fractional order. When $\delta = 0$, it recovers to the elastic material; when $\delta = 1$, it recovers to the Kelvin-Voigt model (a spring and a dashpot in parallel). Inserting Eq. (S13) into Eq. (S1), the wave equation can be expressed by [4]

$$-\hat{q}_{,j} - G\hat{Q}_{,j} + G\mathcal{A}^0_{ijkl}u_{l,ik} - \Omega\sigma^e_{Dik}u_{j,ik} = \rho u_{j,tt}, \quad i,j,k,l \in \{x,y,z\} \tag{S14}$$

The viscoelastic single-layer model consists of a thin plate with a thickness of h, where its top surface in contact with air, and its bottom surface in contact with an inviscid fluid. Following the similar analysis in Supplementary Note 1, we introduce stream function ψ to replace displacements— $u_x = \psi_{,y}$ and $u_y = -\psi_{,x}$. Inserting ψ into Eq. (S14), we obtain the wave equation as follows

$$G(\alpha\psi_{,xxxx} + 2\beta\psi_{,xxyy} + \gamma\psi_{,yyyy}) - \Omega[\sigma^e_{Dxx}\psi_{,xxxx} + \sigma^e_{Dyy}\psi_{,yyyy} + (\sigma^e_{Dxx} + \sigma^e_{Dyy})\psi_{,xxyy}] = \rho(\psi_{,xxtt} + \psi_{,yytt}), \tag{S15}$$

where the explicit forms of acoustoelastic parameters α, β, and γ are given in Supplementary Note 5. The deviatoric elastic stress $\sigma^e_{Dij} = \sigma^e_{ij} - Q$. The elastic stresses are related to the elasticity tensor as follows

$$Q = \frac{\alpha+\gamma+\mathcal{A}^0_{zyzy}}{3},\ \sigma^e_{Dxx} = \frac{2}{3}\alpha - \frac{\gamma}{3} - \frac{\mathcal{A}^0_{zyzy}}{3},\ \sigma^e_{Dyy} = \frac{2}{3}\gamma - \frac{\alpha}{3} - \frac{\mathcal{A}^0_{zyzy}}{3}, \tag{S16}$$

The elastic waves are assumed to propagate along the *x*-axis, therefore the stream function follows the same harmonic form as adopted in the elastic model: $\psi = \psi_0 \exp(sky)\exp[i(kx-\omega t)]$. Inserting the harmonic form of ψ into Eq. (S15) yields

$$(G\gamma - \Omega\sigma^e_{Dyy})s^4 + \left[\rho\frac{\omega^2}{k^2} - 2G\beta + \Omega(\sigma^e_{Dxx} + \sigma^e_{Dyy})\right]s^2 + \left(G\alpha - \Omega\sigma^e_{Dxx} - \rho\frac{\omega^2}{k^2}\right) = 0. \tag{S17}$$

Four roots, denoted as $\pm s_1$ and $\pm s_2$, can be obtained from Eq. (S17). Thus, the general solution of ψ takes the form: $\psi = \sum_{i=-2}^{2}\psi_i\, e^{sign(i)s_i ky} e^{i(kx-\omega t)}$. With the boundary conditions Eq. (S9), and replacing $\boldsymbol{u}$, $\boldsymbol{u}^f$, $\boldsymbol{\Sigma}$ and p^f with ψ and φ, we obtain the secular equation of the viscoelastic single-layer model as follows

$$\det(\mathbf{M}^{\mathrm{v}}_{\mathbf{5\times5}}) = 0 \tag{S18}$$

where the components of the matrix $\mathbf{M}^{\mathrm{v}}_{\mathbf{5\times5}}$ are

$M_{11} = M_{12} = M_{13} = M_{14} = 1, M_{15} = -i\xi,$

$M_{21} = 1 + s_1^2, M_{22} = 1 + s_1^2, M_{23} = 1 + s_2^2, M_{24} = 1 + s_2^2, M_{25} = 0,$

$M_{31} = C_1 s_1 - C_2 s_1^3 - \rho\frac{\omega^2}{k^2}s_1,\ M_{32} = -(C_1 s_1 - C_2 s_1^3 - \rho\frac{\omega^2}{k^2}s_1),$

$$M_{33} = C_1 s_2 - C_2 s_2^3 - \rho \frac{\omega^2}{k^2} s_2,\ M_{34} = -(C_1 s_2 - C_2 s_2^3 - \rho \frac{\omega^2}{k^2} s_2),\ M_{35} = \ i\rho_f \frac{\omega^2}{k^2},$$

$$M_{41} = (1 + s_1^2)\exp(s_1 kh), M_{42} = (1 + s_1^2)\exp(-s_1 kh),$$

$$M_{43} = (1 + s_2^2)\exp(s_2 kh), M_{44} = (1 + s_2^2)\exp(-s_2 kh), M_{45} = 0,$$

$$M_{51} = \left(C_1 s_1 - C_2 s_1^3 - \rho \frac{\omega^2}{k^2} s_1\right)\exp(s_1 kh),\ M_{52} = -\left(C_1 s_1 - C_2 s_1^3 - \rho \frac{\omega^2}{k^2} s_1\right)\exp(-s_1 kh),$$

$$M_{53} = \left(C_1 s_2 - C_2 s_2^3 - \rho \frac{\omega^2}{k^2} s_2\right)\exp(s_2 kh),\ M_{54} = -\left(C_1 s_2 - C_2 s_2^3 - \rho \frac{\omega^2}{k^2} s_2\right)\exp(-s_2 kh),\ M_{55} = 0. \tag{S19}$$

where C_1 and C_2 are two coefficients defined as

$$C_1 = 2G\beta + \gamma + \Omega \mathcal{A}_{zyzy}^0,\ C_2 = \gamma + \frac{1}{3}\Omega(\alpha + \gamma + \mathcal{A}_{zyzy}^0), \tag{S20}$$

The symbol 'i' in M_{15} and M_{35} denotes the imaginary unit. ξ is defined by Eq. (S8). ρ and ρ_f denote the density of the plate and fluid, respectively. $\pm s_1$ and $\pm s_2$ are the roots of Eq. (S17).

One degenerate case can be validated: by inserting $G = 1$ and $\Omega = 0$ into Eq. (S18), the elastic single-layer model (i.e. Eq. (S10)) can be recovered.

Supplementary Note 3. Derivation of the pre-stressed viscoelastic two-layer model

Now we consider a two-layer model, denoted as Layer 1 and Layer 2, with respective thickness h_1 and h_2. The top surface of Layer 1 is exposed to air, while the bottom surface of Layer 2 interfaces with a semi-infinite inviscid fluid. A Cartesian coordinate system (*x*, *y*, *z*) was established on the model, where the *y*-axis denotes the thickness direction, and the *x*- and *z*-axes denote the two directions parallel to the layers. Similar to the analysis in Supplementary Note 2, the stream function in Layer 1 follows a harmonic form of $\psi = \psi_0 \exp(sky)\exp[i(kx - \omega t)]$, and the stream function in Layer 2 follows a harmonic form of $\psi^* = \psi_0^* \exp(s^* ky)\exp[i(kx - \omega t)]$. Inserting ψ into Eq. (S15), the wave equation in Layer 1 satisfies

$$\left(G_1\gamma_1 - \Omega_1\sigma_{Dyy}^{e1}\right)s^4 + \left[\rho_1\frac{\omega^2}{k^2} - 2G_1\beta_1 + \Omega_1(\sigma_{Dxx}^{e1} + \sigma_{Dyy}^{e1})\right]s^2 + \left(G_1\alpha_1 - \Omega_1\sigma_{Dxx}^{e1} - \rho_1\frac{\omega^2}{k^2}\right) = 0. \tag{S21}$$

Inserting ψ^* into Eq. (S14), the wave equation in Layer 2 is

$$\left(G_2\gamma_2 - \Omega_2\sigma_{Dyy}^{e2}\right)s^{*4} + \left[\rho_2\frac{\omega^2}{k^2} - 2G_2\beta_2 + \Omega_2(\sigma_{Dxx}^{e2} + \sigma_{Dyy}^{e2})\right]s^{*2} + \left(G_2\alpha_2 - \Omega_2\sigma_{Dxx}^{e2} - \rho_2\frac{\omega^2}{k^2}\right) = 0. \tag{S22}$$

where ρ_1 and ρ_2 are material density of Layer 1 and Layer 2, respectively. σ_{Dij}^{e1} and σ_{Dij}^{e2} are stress of Layer 1 and Layer 2, respectively. α_1, β_1, γ_1 are acoustoelastic parameters of Layer 1 (related to the elasticity tensor $\mathcal{A}_{ijkl}^0$ of Layer 1). α_2, β_2, γ_2 are acoustoelastic parameters of Layer 2 (related to the elasticity tensor $\mathcal{A}_{ijkl}^{0*}$ of Layer 2). G_1 and Ω_1 are frequency-dependent parameters of Layer 1. G_2 and Ω_2 are frequency-dependent parameters of Layer 2. They are

$$G_1 = 1 + \eta_1(i\omega)^{\delta_1},\ \Omega_1 = \eta_1(i\omega)^{\delta_1},$$
$$G_2 = 1 + \eta_2(i\omega)^{\delta_2},\ \Omega_2 = \eta_2(i\omega)^{\delta_2}, \tag{S23}$$

where η_1 (relative strength of the viscosity compared to the elasticity) and δ_1 (fractional order)

are the KVFD parameters of Layer 1. η_2 and δ_2 are the KVFD parameters of Layer 2.

The interface of the two layers (at $y=0$) ensures continuity of displacement and stress. The surface of Layer 1 exposed to air (at $y=h_1$) satisfies stress-free boundary conditions. The surface of Layer 2 exposed to the fluid (at $y=-h_2$) satisfies the continuity of the normal displacement and stress. These boundary conditions are expressed as

$$
\begin{aligned}
&u_x=u_x^*, u_y=u_y^*, \Sigma_{yx}=\Sigma_{yx}^*, \Sigma_{yy}=\Sigma_{yy}^*, \quad \text{at } y=0 \\
&\Sigma_{yx}=0, \Sigma_{yy}=0, \quad \text{at } y=h_1 \\
&u_y^*=u_y^f, \Sigma_{yx}^*=0, \Sigma_{yy}^*=-p^f, \quad \text{at } y=-h_2
\end{aligned}
\tag{S24}
$$

where u_i, u_i^* and u_i^f denote the displacement of Layer 1, Layer 2 and the fluid, respectively. Σ_{ij} and Σ_{ij}^* denote the incremental stress of Layer 1 and Layer 2, respectively. p^f is the hydrostatic pressure of the fluid. Using ψ, ψ^* and φ to replace displacements and stresses in the boundary conditions Eq. (S24), we obtain the secular equation of the viscoelastic two-layer model as follows

$$
\det(\mathbf{M}_{\mathbf{9}\times\mathbf{9}}^{\mathrm{v}})=0 \tag{S25}
$$

where the components of the matrix $\mathbf{M}_{\mathbf{9}\times\mathbf{9}}^{\mathrm{v}}$ include

$M_{11}=(1+s_1^2)\exp(s_1kh_1), M_{12}=(1+s_2^2)\exp(s_2kh_1)$,

$M_{13}=(1+s_1^2)\exp(-s_1kh_1), M_{14}=(1+s_2^2)\exp(-s_2kh_1), M_{15}=M_{16}=M_{17}=M_{18}=M_{19}=0$,

$M_{21}=\left(C_1s_1-C_2s_1^3-\rho_1\frac{\omega^2}{k^2}s_1\right)\exp(s_1kh_1),\ M_{22}=\left(C_1s_2-C_2s_2^3-\rho_1\frac{\omega^2}{k^2}s_2\right)\exp(s_2kh_1)$,

$M_{23}=-\left(C_1s_1-C_2s_1^3-\rho_1\frac{\omega^2}{k^2}s_1\right)\exp(-s_1kh_1),\ M_{24}=-\left(C_1s_2-C_2s_2^3-\rho_1\frac{\omega^2}{k^2}s_2\right)\exp(-s_2kh_1)$,

$M_{25}=M_{26}=M_{27}=M_{28}=M_{29}=0$,

$M_{31}=M_{32}=M_{33}=M_{34}=0,\ M_{35}=\exp(-s_1^*kh_2),\ M_{36}=\exp(-s_2^*kh_2),\ M_{37}=\exp(s_1^*kh_2)$,

$M_{38}=\exp(s_2^*kh_2),\ M_{39}=-i\xi\exp(-\xi kh_2)$,

$M_{41}=M_{42}=M_{43}=M_{44}=0,\ M_{45}=(1+s_1^{*2})\exp(-s_1^*kh_2),\ M_{46}=(1+s_2^{*2})\exp(-s_2^*kh_2)$,

$M_{47} = (1 + s_1^{*2})\exp(s_1^* k h_2),\ M_{48} = (1 + s_2^{*2})\exp(s_2^* k h_2),\ M_{49} = 0,$

$M_{51} = M_{52} = M_{53} = M_{54} = 0,\ M_{55} = \left(C_1^* s_1^* - C_2^* s_1^{*3} - \rho_2 \frac{\omega^2}{k^2} s_1^*\right)\exp(-s_1^* k h_2),$

$M_{56} = \left(C_1^* s_2^* - C_2^* s_2^{*3} - \rho_2 \frac{\omega^2}{k^2} s_2^*\right)\exp(-s_2^* k h_2),\ M_{57} = -\left(C_1^* s_1^* - C_2^* s_1^{*3} - \rho_2 \frac{\omega^2}{k^2} s_1^*\right)\exp(s_1^* k h_2),$

$M_{58} = -\left(C_1^* s_2^* - C_2^* s_2^{*3} - \rho_2 \frac{\omega^2}{k^2} s_2^*\right)\exp(s_2^* k h_2),\ M_{59} = i\rho_f \frac{\omega^2}{k^2}\exp(-\xi k h_2),$

$M_{61} = s_1, M_{62} = s_2, M_{63} = -s_1, M_{64} = -s_2, M_{65} = -s_1^*, M_{66} = -s_2^*, M_{67} = s_1^*, M_{68} = s_2^*, M_{69} = 0,$

$M_{71} = M_{72} = M_{73} = M_{74} = 1, M_{75} = M_{76} = M_{77} = M_{78} = -1, M_{79} = 0,$

$M_{81} = C_2(1 + s_1^2), M_{82} = C_2(1 + s_2^2), M_{83} = C_2(1 + s_1^2), M_{84} = C_2(1 + s_2^2), M_{85} = -C_2^*(1 + s_1^{*2}),$

$M_{86} = -C_2^*(1 + s_2^{*2}), M_{87} = -C_2^*(1 + s_1^{*2}), M_{88} = -C_2^*(1 + s_2^{*2}), M_{89} = 0,$

$M_{91} = C_1 s_1 - C_2 s_1^3 - \rho_1 \frac{\omega^2}{k^2} s_1,\ M_{92} = C_1 s_2 - C_2 s_2^3 - \rho_1 \frac{\omega^2}{k^2} s_2,\ M_{93} = -(C_1 s_1 - C_2 s_1^3 - \rho_1 \frac{\omega^2}{k^2} s_1),$

$M_{94} = -(C_1 s_2 - C_2 s_2^3 - \rho_1 \frac{\omega^2}{k^2} s_2),\ M_{95} = -(C_1^* s_1^* - C_2^* s_1^{*3} - \rho_2 \frac{\omega^2}{k^2} s_1^*),$

$M_{96} = -(C_1^* s_2^* - C_2^* s_2^{*3} - \rho_2 \frac{\omega^2}{k^2} s_2^*),\ M_{97} = C_1^* s_1^* - C_2^* s_1^{*3} - \rho_2 \frac{\omega^2}{k^2} s_1^*,$

$M_{98} = C_1^* s_2^* - C_2^* s_2^{*3} - \rho_2 \frac{\omega^2}{k^2} s_2^*,\ M_{99} = 0.$ (S26)

where i in the element M_{39} and M_{59} denotes the imaginary unit. ξ is define by Eq. (S8). ρ_1, ρ_2, and ρ_f denote the density of Layer 1, Layer 2, and the fluid, respectively. $\pm s_1$ and $\pm s_2$ are the roots solved by Eq. (S21). $\pm s_1^*$ and $\pm s_2^*$ are the roots solved by Eq. (S22). Coefficients C_1, C_2, C_1^*, and C_2^* are defined by

$$C_1 = 2G_1\beta_1 + \gamma_1 + \Omega_1 \mathcal{A}^0_{zyzy},\ C_2 = \gamma_1 + \frac{1}{3}\Omega_1(\alpha_1 + \gamma_1 + \mathcal{A}^0_{zyzy}),$$
$$C_1^* = 2G_2\beta_2 + \gamma_2 + \Omega_2 \mathcal{A}^{0*}_{zyzy},\ C_2^* = \gamma_2 + \frac{1}{3}\Omega_2(\alpha_2 + \gamma_2 + \mathcal{A}^{0*}_{zyzy}). \quad \text{(S27)}$$

One degenerate case can be validated: by substituting $h = h_1 + h_2$ and assuming identical material properties for the two layers (i.e. $\alpha_1 = \alpha_2,\ \gamma_1 = \gamma_2,\ \beta_1 = \beta_2,\ \eta_1 = \eta_2,\ \delta_1 = \delta_2,\ \rho_1 = \rho_2$, etc.) into Eq. (S25), the viscoelastic single-layer model given in Eq. (S18) is recovered.

Supplementary Note 4. Pre-stressed elastic two-layer model

The elastic two-layer guided wave model can be obtained as a special case of the viscoelastic two-layer model by inserting $G_1 = G_2 = 1$ and $\Omega_1 = \Omega_2 = 0$ into Eq. (S25), which yields

$$\det(\mathbf{M}^{\mathrm{e}}_{\mathbf{9}\times\mathbf{9}}) = 0 \tag{S28}$$

where the nonzero components of the matrix $\mathbf{M}^{\mathrm{e}}_{\mathbf{9}\times\mathbf{9}}$ include

$M_{11} = (1+s_1^2)\exp(s_1kh_1), M_{12} = (1+s_2^2)\exp(s_2kh_1), M_{13} = (1+s_1^2)\exp(-s_1kh_1),$

$M_{14} = (1+s_2^2)\exp(-s_2kh_1),$

$M_{21} = s_1(1+s_2^2)\exp(s_1kh_1), M_{22} = s_2(1+s_1^2)\exp(s_2kh_1),$

$M_{23} = -s_1(1+s_2^2)\exp(-s_1kh_1), M_{24} = -s_2(1+s_1^2)\exp(-s_2kh_1),$

$M_{35} = \exp(-s_1^*kh_2), M_{36} = \exp(-s_2^*kh_2), M_{37} = \exp(s_1^*kh_2), M_{38} = \exp(s_2^*kh_2),$

$M_{39} = -i\xi\exp(-\xi kh_2),$

$M_{45} = (1+s_1^{*2})\exp(-s_1^*kh_2), M_{46} = (1+s_2^{*2})\exp(-s_2^*kh_2),$

$M_{47} = (1+s_1^{*2})\exp(s_1^*kh_2), M_{48} = (1+s_2^{*2})\exp(s_2^*kh_2),$

$M_{55} = \gamma_2 s_1^*(1+s_2^{*2})\exp(-s_1^*kh_2), M_{56} = \gamma_2 s_2^*(1+s_1^{*2})\exp(-s_2^*kh_2),$

$M_{57} = -\gamma_2 s_1^*(1+s_2^{*2})\exp(s_1^*kh_2), M_{58} = -\gamma_2 s_2^*(1+s_1^{*2})\exp(s_2^*kh_2),$

$M_{59} = -i\rho_f\exp(-\xi kh_2)\omega^2/k^2,$

$M_{61} = s_1, M_{62} = s_2, M_{63} = -s_1, M_{64} = -s_2, M_{65} = -s_1^*, M_{66} = -s_2^*, M_{67} = s_1^*, M_{68} = s_2^*,$

$M_{71} = M_{72} = M_{73} = M_{74} = 1, M_{75} = M_{76} = M_{77} = M_{78} = -1,$

$M_{81} = \gamma_1(1+s_1^2), M_{82} = \gamma_1(1+s_2^2), M_{83} = \gamma_1(1+s_1^2), M_{84} = \gamma_1(1+s_2^2),$

$M_{85} = -\gamma_2(1+s_1^{*2}), M_{86} = -\gamma_2(1+s_2^{*2}), M_{87} = -\gamma_2(1+s_1^{*2}), M_{88} = -\gamma_2(1+s_2^{*2}),$

$M_{91} = \gamma_1 s_1(1+s_2^2), M_{92} = \gamma_1 s_2(1+s_1^2), M_{93} = -\gamma_1 s_1(1+s_2^2), M_{94} = -\gamma_1 s_2(1+s_1^2),$

$$M_{95}=-\gamma_2 s_1^*(1+s_2^{*2}), M_{96}=-\gamma_2 s_2^*(1+s_1^{*2}), M_{97}=\gamma_2 s_1^*(1+s_2^{*2}), M_{98}=\gamma_2 s_2^*(1+s_1^{*2}). \quad \text{(S29)}$$

where $\pm s_1$ and $\pm s_2$ are the four roots solved by the quartic equation

$$\gamma_1 s^4-\left(2\beta_1-\rho_1\frac{\omega^2}{k^2}\right)s^2+\alpha_1-\rho_1\frac{\omega^2}{k^2}=0 \quad \text{(S30)}$$

$\pm s_1^*$ and $\pm s_2^*$ are the four roots solved by the quartic equation

$$\gamma_2 {s^*}^4-\left(2\beta_2-\rho_2\frac{\omega^2}{k^2}\right){s^*}^2+\alpha_2-\rho_2\frac{\omega^2}{k^2}=0 \quad \text{(S31)}$$

ρ_1, ρ_2 and ρ_f are material density of the Layer 1, Layer 2 and fluid, respectively. α_1, γ_1 and β_1 are acoustoelastic parameters of Layer 1. α_2, γ_2 and β_2 are acoustoelastic parameters of Layer 2. ξ is defined in Eq. (S8).

Supplementary Note 5. Explicit forms of the acoustoelastic parameters

Gasser-Ogden-Holzapfel (GOH) constitutive model has been widely adopted to describe arterial hyperelasticity [7]. As shown in Fig. S4, we cut the tube longitudinally and unfold it into a flat plate. A Cartesian coordinate system (x_r, x_c, x_a) is established on the plate, representing the radial (depth direction), circumferential, and axial direction of the artery, respectively (Fig. S4b). The strain energy function is

$$W = \frac{\mu_0}{2}(I_1 - 3) + \frac{k_1}{2k_2}\sum_{i=4,6}\{\exp[k_2(\kappa I_1 + (1-3\kappa)I_i - 1)^2] - 1\} \tag{S32}$$

The first term on the right-hand side of Eq. (S32) describes the isotropic elastin matrix. The second term describes anisotropic collagen fibers. μ_0 denotes the matrix shear modulus. k_1 represents the collagen fiber-related shear modulus. k_2 is a dimensionless parameter denoting the nonlinear hardening effect of the collagen fibers. κ is a fiber dispersion parameter, ranging from 0 for highly organized fibers to 1/3 random isotropic orientations. The first principle invariant $I_1 = \mathrm{tr}(\boldsymbol{F}^\mathrm{T}\boldsymbol{F})$, where $\boldsymbol{F}$ is the deformation gradient tensor. I_4 and I_6 are two invariants related to two families of collagen fibers arranged along the preferred directions, $\boldsymbol{m}_1$ and $\boldsymbol{m}_2$, respectively. $I_4 = \boldsymbol{Fm_1} \cdot \boldsymbol{Fm_1}$ and $I_6 = \boldsymbol{Fm_2} \cdot \boldsymbol{Fm_2}$. This model assumes that the two families of collagen fibers are symmetrically distributed within the plane (Fig. S4), with orientations $\boldsymbol{m}_1 = (0, \cos\varphi, \sin\varphi)^\mathrm{T}$, and $\boldsymbol{m}_2 = (0, -\cos\varphi, \sin\varphi)^\mathrm{T}$, where φ is the angle between the fiber orientations and the circumferential direction. Therefore $I_4 = I_6 = I'$, and the second term on the right-hand side of Eq. (S32) can be rewritten as a function of I', resulting in the form shown in Eq. (10) in the main text.

Applying the above strain energy function into the definition of elasticity tensor $\mathcal{A}^0_{ijkl}$ (i.e. Eq. S3), we derive the explicit forms of the acoustoelastic parameters as follows:

$$\alpha_a = 2W_1{\lambda_a}^2 + 2W_4{\lambda_a}^2\sin^2\varphi + 2W_6{\lambda_a}^2\sin^2\varphi$$

$$\begin{aligned}\beta_a = W_1\left({\lambda_a}^2 + {\lambda_r}^2\right) + W_4{\lambda_a}^2\sin^2\varphi + W_6{\lambda_a}^2\sin^2\varphi + 2W_{11}\left({\lambda_a}^2 - {\lambda_r}^2\right)^2 \\ + 4W_{14}{\lambda_a}^2\sin^2\varphi\left({\lambda_a}^2 - {\lambda_r}^2\right) + 4W_{16}{\lambda_a}^2\sin^2\varphi\left({\lambda_a}^2 - {\lambda_r}^2\right) \\ + 2W_{44}{\lambda_a}^4\sin^4\varphi + 2W_{66}{\lambda_a}^4\sin^4\varphi\end{aligned}$$

$$\alpha_c = 2W_1{\lambda_c}^2 + 2W_4{\lambda_c}^2\cos^2\varphi + 2W_6{\lambda_c}^2\cos^2\varphi \qquad \text{(S33)}$$

$$\begin{aligned}\beta_c = W_1\left({\lambda_c}^2 + {\lambda_r}^2\right) + W_4{\lambda_c}^2\cos^2\varphi + W_6{\lambda_c}^2\cos^2\varphi + 2W_{11}\left({\lambda_c}^2 - {\lambda_r}^2\right)^2 \\ + 4W_{14}{\lambda_c}^2\cos^2\varphi\left({\lambda_c}^2 - {\lambda_r}^2\right) + 4W_{16}{\lambda_c}^2\cos^2\varphi\left({\lambda_c}^2 - {\lambda_r}^2\right) \\ + 2W_{44}{\lambda_c}^4\cos^4\varphi + 2W_{66}{\lambda_c}^4\cos^4\varphi\end{aligned}$$

$$\gamma_a = \gamma_c = 2W_1{\lambda_r}^2$$

where α_a $(= \mathcal{A}^0_{arar})$, β_a $(= (\mathcal{A}^0_{aaaa} + \mathcal{A}^0_{rrrr} - 2\mathcal{A}^0_{aarr} - 2\mathcal{A}^0_{arra})/2)$ and γ_a $(= \mathcal{A}^0_{rara})$ correspond to the acoustoelastic parameters along the axial direction of the artery. α_c $(= \mathcal{A}^0_{crcr})$, β_c $(= (\mathcal{A}^0_{cccc} + \mathcal{A}^0_{rrrr} - 2\mathcal{A}^0_{ccrr} - 2\mathcal{A}^0_{crrc})/2)$ and γ_c $(= \mathcal{A}^0_{rcrc})$ are acoustoelastic parameters along the circumferential direction of the artery. λ_r, λ_c, λ_a denote radial, circumferential, and axial stretch ratio, respectively. $W_i = \partial W/\partial I_i$, $W_{ij} = \partial^2 W/\partial I_i\,\partial I_j$, where $i, j = 1, 4, 6$. $I_1 = {\lambda_c}^2 + {\lambda_r}^2 + {\lambda_a}^2$. $I_4 = I_6 = {\lambda_c}^2\cos^2\varphi + {\lambda_a}^2\sin^2\varphi$. The parameter $\mathcal{A}^0_{zyzy}$ used in Eqs. (S20) and (S27) is equal to α_c when along the axial direction, while equal to α_a when along the circumferential direction.

In the stress-free state ($\lambda_c = \lambda_r = \lambda_a = 1$), Eq. (S33) reduces to $\alpha_a = \alpha_c = \mu_0$, $2\beta_a + 2\gamma_a = 4\mu_0 + 8k_1(1 - 3\kappa)^2\sin^2\varphi$, and $2\beta_c + 2\gamma_c = 4\mu_0 + 8k_1(1 - 3\kappa)^2\cos^2\varphi$. Therefore we have $(2\beta + 2\gamma)/\alpha > 4$ when $\kappa < 1/3$. Since the ratio $(2\beta + 2\gamma)/\alpha = 4$ corresponds to isotropy, and higher values indicate greater anisotropy [8], this result again suggests the anisotropic nature of arteries.

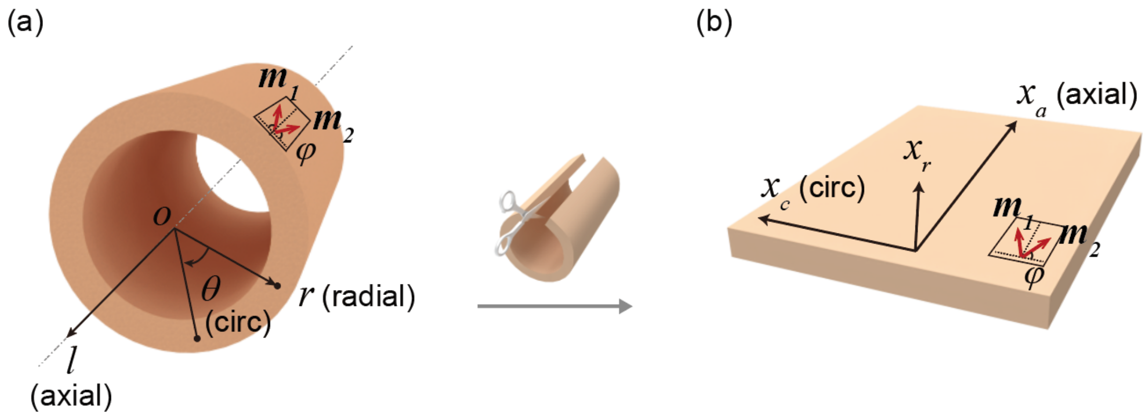

Figure S4. Schematic of the Gasser-Ogden-Holzapfel constitutive model. (a) A tube-shaped artery and (b) square arterial tissue sample with orientation axes and unit vectors of the two fiber families. $\boldsymbol{m}_1$ and $\boldsymbol{m}_2$ indicate orientation of the two symmetric fiber families, which induces the anisotropy of arteries.

Supplementary Note 6. In-plane tensile modulus of a static plate

In the following, we consider a plate that is infinite in the x_3 direction, with x_1 as the longitudinal direction and x_2 as the thickness direction. After a finite pre-stretch, the plate further undergoes an incremental in-plane uniaxial tensile deformation along the x_1 direction. The following conditions are satisfied: displacement $u_3 = 0$, the normal stress $\Sigma_{22} = 0$, shear stress and shear strain components vanish, and all field variables are independent of the x_3 direction. With the above assumptions and the elastic stress-strain relation given in Eq. (S2), the incremental stresses are related to the displacements as follows:

$$\Sigma_{11} = \mathcal{A}^0_{1111}u_{1,1} + \mathcal{A}^0_{1122}u_{2,2} - \hat{p} + pu_{1,1} \tag{S34}$$

$$\Sigma_{22} = \mathcal{A}^0_{1122}u_{2,2} + \mathcal{A}^0_{2222}u_{2,2} - \hat{p} + pu_{2,2} \tag{S35}$$

The incompressible condition is $u_{1,1} + u_{2,2} = 0$. Using the stress assumption $\Sigma_{22} = 0$ into Eq. (S35), together with the incompressible relation, we can rewrite Σ_{11} into the following form:

$$\Sigma_{11} = (\mathcal{A}^0_{1111} + \mathcal{A}^0_{2222} - 2\mathcal{A}^0_{1122} + 2p)u_{1,1} \tag{S36}$$

Since $\sigma_{22} = 0$ (Cauchy stress in the deformed state), the Lagrange multiplier p is related to the acoustoelastic parameters as follows [2]

$$p = \mathcal{A}^0_{2121} - \mathcal{A}^0_{1221} \tag{S37}$$

Substituting Eq. (S37) into Eq. (S36), we can rewrite Σ_{11} as

$$\Sigma_{11} = (\mathcal{A}^0_{1111} + \mathcal{A}^0_{2222} - 2\mathcal{A}^0_{1122} + 2\mathcal{A}^0_{2121} - 2\mathcal{A}^0_{1221})u_{1,1} \tag{S38-a}$$

The above equation is equivalent to

$$\Sigma_{11} = (2\beta + 2\gamma)u_{1,1} \tag{S38-b}$$

Eq. (S38-b) demonstrates the relationship between tensile stress and strain; therefore, $2\beta + 2\gamma$ corresponds to the in-plane tensile modulus.

Supplementary Note 7. Complex dynamic modulus in pre-stressed viscoelastic materials

In viscoelastic materials, the shear wave velocity is determined by the complex shear modulus

$$\rho\frac{\omega^2}{k^2} = \mu^* \tag{S39}$$

Inserting $s = 0$ into wave equation Eq. (S17), we can obtain μ^* as follows

$$\mu^* = G\alpha - \Omega\sigma^e_{Dxx} = G\alpha - \frac{1}{3}\Omega\left(2\alpha - \gamma - \mathcal{A}^0_{zyzy}\right) \tag{S40}$$

The plate wave velocity is determined by the complex tensile modulus

$$\rho\frac{\omega^2}{k^2} = \bar{E}^* \tag{S41}$$

In order to obtain the explicit form of $\bar{E}^*$ in pre-stressed viscoelastic material, we make use of incremental stress-strain relation given in Eq. (S12), and by following a similar derivation as shown in Supplementary Note 6, we get a relation $\Sigma_{xx} = \bar{E}^* u_{x,x}$ and

$$\bar{E}^* = G(2\beta + 2\gamma) - \Omega\left(\sigma^e_{Dxx} + \sigma^e_{Dyy} + 2q\right) = G(2\beta + 2\gamma) - \frac{1}{3}\Omega\left(-\alpha + 5\gamma - 4\mathcal{A}^0_{zyzy}\right) \tag{S42}$$

Notably, unlike the linear case, the complex shear modulus is not simply $\mu^* = G\alpha$, and the complex tensile modulus is not directly $\bar{E}^* = G(2\beta + 2\gamma)$. This is primarily because, in the incremental dynamics framework, the material is first subjected to a fully relaxed finite pre-stress, and then viscoelastic wave motions are superimposed on top of it. As a result, some long-term Cauchy stress terms are retained in Eqs. (S40) and (S42).

Figure S5a compares the complex shear modulus μ^* and its first term $G\alpha$. The material parameters are based on the fitting results from experimental data. As shown, both the real and imaginary parts of μ^* are close to those of $G\alpha$ (relative error < 3% for the real part, and <14% for the imaginary part). Figure S5b compares the complex tensile modulus $\bar{E}^*$ and its first term $G(2\beta + 2\gamma)$. The relative difference between $\bar{E}^*$ and $G(2\beta + 2\gamma)$ is below 6% for the real part,

and below 20% for the imaginary part. Therefore, in this study, it is reasonable to use $\mu^* \approx G\alpha$ and $\bar{E}^* \approx G(2\beta + 2\gamma)$ to approximate complex shear and tensile moduli, respectively, and we use these formulations to demonstrate how viscoelastic effects diminish with increasing pre-stress (as shown in Figs. 3 and 7).

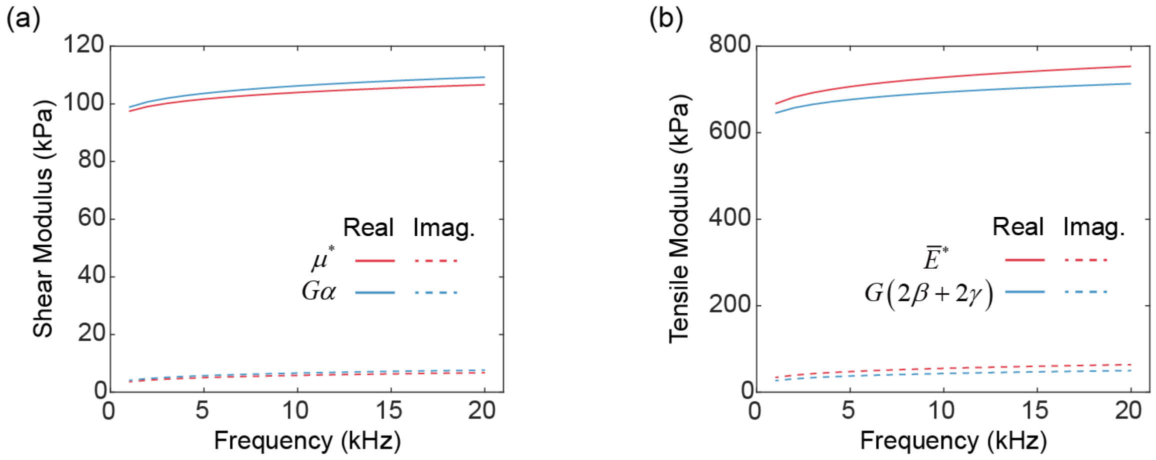

Figure S5. Comparison of complex moduli and their approximate forms. (a) Complex shear modulus μ^* and its first term $G\alpha$. (b) Complex tensile modulus $\bar{E}^*$ and its first term $G(2\beta + 2\gamma)$. The material parameters used here are obtained from the fitting results along the axial direction at $\lambda = 1.2$ (see Table 2), including $\alpha = 87$ kPa, $\gamma = 21$ kPa, $\beta = 263$ kPa, $\eta = 0.0023$, $\delta = 0.21$.

Supplementary Note 8. Asymptotic phase velocities of the A0 and S0 modes at high frequencies in the two-layer model

S8.1 Asymptotic solutions and their implication for experimental measurement

Figure S6 shows a schematic of the two-layer viscoelastic model, and representative dispersion curves of the A0 and S0 modes. We denote the high-frequency asymptotic phase velocities of the A0 and S0 modes as $c_{A0,\mathrm{asymp}}$ and $c_{S0,\mathrm{asymp}}$, respectively. They can be generally expressed by

$$c_{A0,\mathrm{asymp}} = \min\{c_{R1}, c_{S2}\} \tag{S43}$$

$$c_{S0,\mathrm{asymp}} = \text{second smallest of } \{c_{R1}, c_{t1}, c_{S2}, c_{t2}\} \tag{S44}$$

where c_R, c_S and c_t denote the Rayleigh surface wave, Scholte wave (fluid-solid interface wave), and plane shear wave velocity. The subscript i (= 1, 2) denotes Layer i. For pre-stressed elastic materials, we denote $c_{R1} = n_{R1}c_{t1}$, and $c_{S2} = n_{S2}c_{t2}$, where n_S and n_R are dimensionless parameters less than 1. Based on the criterion given by Eqs. (S43) and (S44), the results can be divided into four regions according to the shear modulus ratio of the two layers (α_2/α_1), with each region corresponding to distinct asymptotic solutions:

- Region A: $\frac{\alpha_2}{\alpha_1} < {n_{R1}}^2$

$$c_{A0,\mathrm{asymp}} = n_{S2}c_{t2},\ \ c_{S0,\mathrm{asymp}} = c_{t2}$$

- Region B: ${n_{R1}}^2 \le \frac{\alpha_2}{\alpha_1} < \left(\frac{n_{R1}}{n_{S2}}\right)^2$

$$c_{A0,\mathrm{asymp}} = n_{S2}c_{t2},\ \ c_{S0,\mathrm{asymp}} = n_{R1}c_{t1}$$

- Region C: $\left(\frac{n_{R1}}{n_{S2}}\right)^2 \le \frac{\alpha_2}{\alpha_1} < \left(\frac{1}{n_{S2}}\right)^2$

$$c_{A0,\mathrm{asymp}} = n_{R1}c_{t1},\ \ c_{S0,\mathrm{asymp}} = n_{S2}c_{t2}$$

- Region D: $\frac{\alpha_2}{\alpha_1} \ge \left(\frac{1}{n_{S2}}\right)^2$

$$c_{A0,\mathrm{asymp}} = n_{R1}c_{t1},\ \ c_{S0,\mathrm{asymp}} = c_{t1}$$

For linear elastic and isotropic materials, $n_S = 0.839$ and $n_R = 0.955$, and the ranges of the four

regions reduce to $\mu_2/\mu_1 \in (0, 0.912)$, $[0.912, 1.296)$, $[1.296,\ 1.421)$, and $[1.421, +\infty)$, respectively. For pre-stressed elastic materials, n_R and n_S can be determined by solving the following two equations. The Rayleigh wave equation in the pre-stressed elastic material is [9]

$$s_1(1+{s_2}^2)^2 - s_2(1+{s_1}^2)^2 = 0 \tag{S45}$$

and the Scholte wave equation is [10]

$$\gamma s_2(1+{s_1}^2)^2 - \gamma s_1(1+{s_2}^2)^2 + ({s_2}^2 - {s_1}^2)\frac{\rho_f}{\xi}\frac{\omega^2}{k^2} = 0 \tag{S46}$$

where ξ is defined in Eq. (S8). s_2 and s_2 are roots solved by Eq. (S6). ρ_f is the fluid density. Eqs. (S45) and (S46) indicate that n_S and n_R are functions of the acoustoelastic parameters α, γ, and β. It can be shown that n_R increase with the stretch ratio and approaches 1. The variation of n_S is model-dependent; for the GOH model, it also increases with increasing stretch.

By fitting to the experimental dispersion, we have obtained the acoustoelastic parameters α_i, γ_i, and β_i $(i = 1, 2)$ at multiple stretch ratios ($\lambda = 1{\sim}1.4$). With the help of Eqs. (S45) – (S46), the values of n_{S2} and n_{R1} at each stretching state can be solved, and then the boundaries of the four regions (i.e. values of ${n_{R1}}^2$, $(n_{R1}/n_{S2})^2$, and $1/{n_{R1}}^2$) at the corresponding stretching state can be determined. The results are shown in Fig. S7. For both axial and circumferential directions, when $\lambda = 1$, the shear modulus ratio of the two layers (α_2/α_1) falls within Region A. When $\lambda \geq 1.1$, the ratios shift into Region D. Therefore, in the stress-free state, the asymptotic solutions of A0 and S0 modes are governed by the adventitia, with $c_{A0,\mathrm{asymp}} = n_{S2}c_{t2}$ and $c_{S0,\mathrm{asymp}} = c_{t2}$, whereas after stretching, the asymptotic solutions are governed by the media, with $c_{A0,\mathrm{asymp}} = n_{R1}c_{t1}$, and $c_{S0,\mathrm{asymp}} = c_{t1}$.

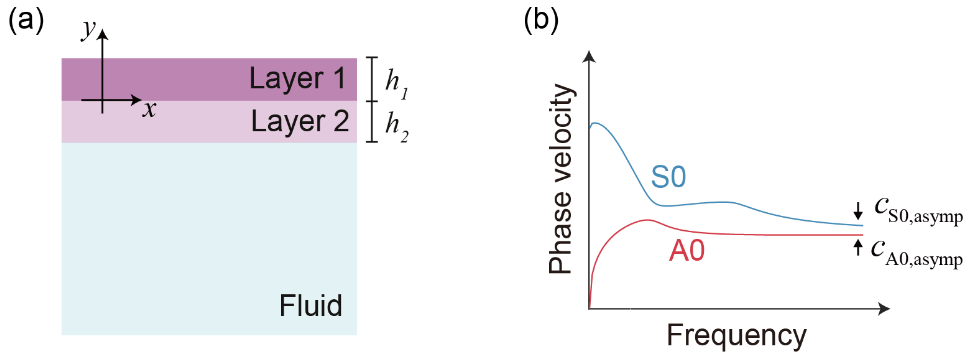

Figure S6. Two-layer guided wave model. (a) Schematic of the model. (b) A0 and S0 modes of the two-layer viscoelastic model. $c_{A0,\mathrm{asymp}}$ and $c_{S0,\mathrm{asymp}}$ denote the high-frequency asymptotic solutions for the two modes.

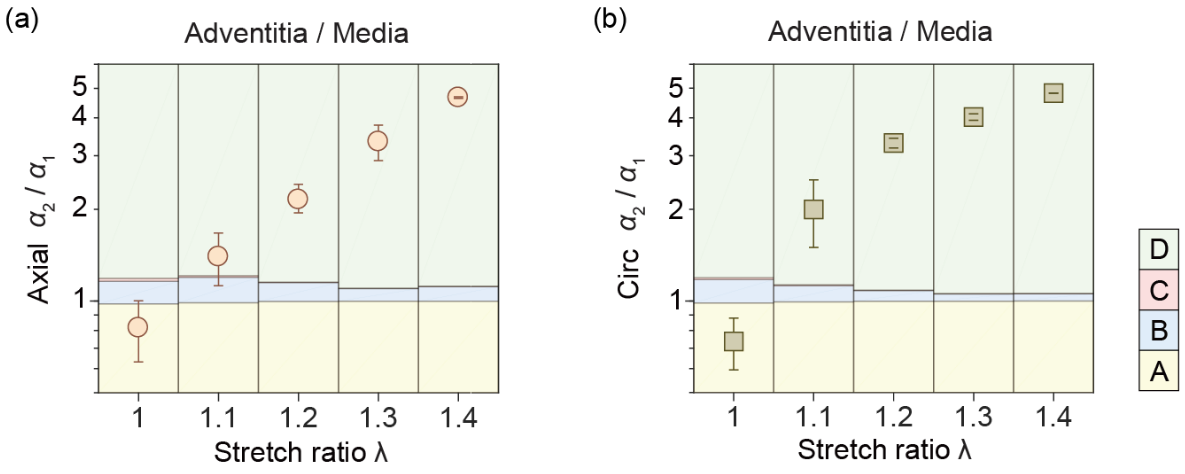

Figure S7. Plot of shear modulus ratio (α_2/α_1) versus stretch ratios, and classification of the four regions at the corresponding stretch ratio. (a) Axial data, (b) Circumferential data.

S8.2 Critical frequencies for the asymptotic velocities of the A0 and S0 modes

The critical frequencies of the asymptotic velocities for the A0 and S0 modes are denoted as $f_{c,A0}$, and $f_{c,S0}$, and they can be approximately estimated as follows:

- Region A: $f_{c,A0} = \frac{c_{S2}}{h_2}$, $f_{c,S0} = \frac{2.5c_{t2}}{h_2}$
- Region B: $f_{c,A0} = \frac{2c_{S2}}{h_1+h_2}$, $f_{c,S0} = \frac{2c_{R1}}{h_1+h_2}$
- Region C: $f_{c,A0} = \frac{c_{R1}}{h_1}$, $f_{c,S0} = \frac{2.5c_{S2}}{h_2}$
- Region D: $f_{c,A0} = \frac{c_{R1}}{h_1}$, $f_{c,S0} = \frac{2.5c_{t1}}{h_1}$

Taking the axial data for $\lambda = 1.4$, for example, the shear modulus of the media is $\alpha_1 = 66\ \text{kPa}$, and $c_{t1} = 8.2\ \text{m/s}$. The wall thickness is $h_1 = 0.3\ \text{mm}$. Using the critical frequencies defined in Region D, we get $f_{c,A0} = 25\ \text{kHz}$, and $f_{c,S0} = 65\ \text{kHz}$. Especially, $f_{c,S0}$ is much higher than 20 kHz, indicating that the current OCE only captures the intermediate frequency range of S0 mode. Higher-frequency measurements may reveal more mechanical information of artery samples.

Supplementary Note 9. Literature data of shear and tensile moduli of artery tissues

We compare our characterization results of the shear and tensile moduli of arterial samples with those reported in the literature. The shear and tensile moduli under different stretch ratios were calculated based on the constitutive parameters of arteries reported in the literature. Figure S8a presents predictions derived from the data of Giudici et al [11], and Fig. S8b shows predictions based on the data of Sommer et al [12]. Figure S8-a1 and b1 shows the bidirectional shear moduli of the media and the adventitia. Figure S8-a2 and b2 shows the bidirectional tensile moduli of the media and the adventitia. In general, these moduli increase with respect to the stretch ratio. The circumferential moduli are higher than the axial ones. Figure S8-a3 and b3 plot the ratio of shear modulus of the two layers. Figure S8-a4 and b4 plot the ratio of tensile modulus of the two layers. These modulus ratios are lower than 1 when the artery is stress-free, indicating a stiffer media in this state. With the increase of the stretch ratio, these ratios increase gradually and surpass 1, indicating that the adventitia becomes significantly stiffer under stretching. These result align well with our experimental results.

The moduli or modulus ratios predicted in the literature at higher stretch ratios (e.g., >1.2) are significantly larger than the values obtained from our experimental fitting. In fact, the stiffening coefficient of collagen fibers reported in the literature is typically above 10, whereas our fitting results yield a fiber stiffening coefficient (k_2) of only around 4. One possible explanation is that the stretch ratio measured in our experiments represents the average value over a broader sample area compared to the area of wave propagation. The localized stretch ratio in the regions where elastic waves propagate could be smaller. The overestimation of the stretch ratio results in an underestimation of the fiber stiffening coefficient.

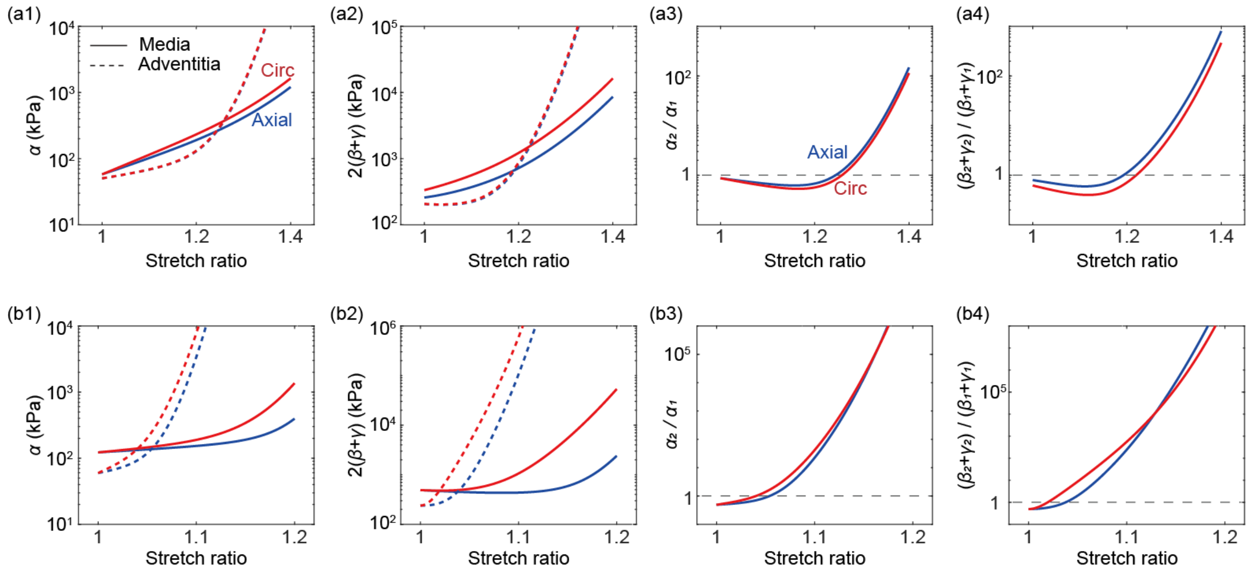

Figure S8. Literature data of shear and tensile moduli of the artery tissues. (a1) - (a4): constitutive parameters obtained from Giudici et al [11]. Tested tissue: porcine thoracic aortas. (b1) – (b4): constitutive parameters obtained from Sommer et al [12]. Tested tissue: human carotid arteries. (a1) and (b1), Bidirectional and bilayers' shear moduli with respect to the stretch ratio. (a2) and (b2), Bidirectional and bilayers' tensile moduli with respect to the stretch ratio. (a3) and (b3), Bidirectional ratios of the shear moduli of the two layers. (a4) and (b4), Bidirectional ratios of the tensile moduli of the two layers.

Supplementary Tables

Table S1. Measured modulus parameters from the two-layer elastic model (1-20 kHz)

		λ = 1.0	λ = 1.1	λ = 1.2	λ = 1.3	λ = 1.4
Axial, intima-media	α (kPa)	94 ± 4	97 ± 5	102 ± 10	116 ± 9	118 ± 8
	$2\beta + 2\gamma$ (kPa)	600 ± 180	630 ± 60	680 ± 150	750 ± 100	1050 ± 140
Axial, adventitia	α (kPa)	82 ± 10	89 ± 9	130 ± 20	170 ± 80	190 ± 35
	$2\beta + 2\gamma$ (kPa)	580 ± 140	890 ± 120	1250 ± 300	2050 ± 350	3200 ± 450
Circum., intima-media	α (kPa)	96 ± 1	140 ± 12	145 ± 8	165 ± 7	190 ± 1
	$2\beta + 2\gamma$ (kPa)	1030 ± 100	1200 ± 100	1380 ± 120	-	-
Circum., adventitia	α (kPa)	80 ± 10	170 ± 40	190 ± 60	260 ± 60	350 ± 20
	$2\beta + 2\gamma$ (kPa)	870 ± 80	1200 ± 520	1950 ± 220	-	-

Reference

1. Ogden, R. W. Incremental statics and dynamics of pre-stressed elastic materials. In: *Waves in nonlinear pre-stressed materials*. Springer (2007).
2. Destrade, M. Incremental equations for soft fibrous materials. In: *Nonlinear mechanics of soft fibrous materials*. Springer (2015).
3. Li, G.-Y. et al. Guided waves in pre-stressed hyperelastic plates and tubes: Application to the ultrasound elastography of thin-walled soft materials. *J. Mech. Phys. Solids* **102**, 67-79 (2017).
4. Jiang, Y., Li, G.-Y., Zhang, Z., Ma, S., Cao, Y. & Yun, S.-H. Incremental dynamics of prestressed viscoelastic solids and its applications in shear wave elastography. *Int. J. Eng. Sci.* **215**, 104310 (2025).
5. Parker, K., Szabo, T. & Holm, S. Towards a consensus on rheological models for elastography in soft tissues. *Phys. Med. Biol.* **64**, 215012 (2019).
6. Bonfanti, A., Kaplan, J. L., Charras, G. & Kabla, A. Fractional viscoelastic models for power-law materials. *Soft Matter* **16**, 6002-6020 (2020).
7. Gasser, T. C., Ogden, R. W. & Holzapfel, G. A. Hyperelastic modelling of arterial layers with distributed collagen fibre orientations. *J. R. Soc. Interface* **3**, 15-35 (2006).
8. Li, G.-Y., Feng, X. & Yun, S.-H. Simultaneous tensile and shear measurement of the human cornea in vivo using S0-and A0-wave optical coherence elastography. *Acta Biomater.* **175**, 114-122 (2024).
9. Dowaikh, M. & Ogden, R. On surface waves and deformations in a pre-stressed incompressible elastic solid. *IMA J. Appl. Math.* **44**, 261-284 (1990).
10. Otténio, M., Destrade, M. & Ogden, R. W. Acoustic waves at the interface of a pre-stressed incompressible elastic solid and a viscous fluid. *Int. J. Non-Linear Mech.* **42**, 310-320 (2007).
11. Giudici, A. & Spronck, B. The role of layer-specific residual stresses in arterial mechanics: analysis via a novel modelling framework. *Artery Res.* **28**, 41-54 (2022).
12. Sommer, G. & Holzapfel, G. A. 3D constitutive modeling of the biaxial mechanical response of intact and layer-dissected human carotid arteries. *J. Mech. Behav. Biomed. Mater.* **5**, 116-128 (2012).
